# Headache Worsening after COVID-19 Vaccination: An Online Questionnaire-Based Study on 841 Patients with Migraine

**DOI:** 10.3390/jcm10245914

**Published:** 2021-12-16

**Authors:** Marcello Silvestro, Alessandro Tessitore, Ilaria Orologio, Pasquale Sozio, Giuseppe Napolitano, Mattia Siciliano, Gioacchino Tedeschi, Antonio Russo

**Affiliations:** 1 Headache Center, Department of Advanced Medical and Surgical Sciences (DAMS), University of Campania “Luigi Vanvitelli”, 80138 Naples, Italy; marcello.silvestro6@gmail.com (M.S.); Alessandro.tessitore@unicampania.it (A.T.); Ilaria.orologio@hotmail.it (I.O.); pakisozio96@gmail.com (P.S.); matsic@hotmail.it (M.S.); gioacchino.tedeschio@unicampania.it (G.T.); 2 Intensity Care Unit, Department of Emergency and Acceptance “Antonio Cardarelli” Hospital, 80131 Naples, Italy; g.napolitano1988@libero.it

**Keywords:** migraine with aura, migraine without aura, COVID-19, vaccine, headache

## Abstract

Vaccines have represented the breakthrough in the fight against COVID-19. Based on reported headache attacks after vaccination in randomized controlled trials, we focused on the effects of COVID-19 vaccine administration on the migraine population, using an online questionnaire published on Italian Facebook groups oriented to headache patients. We collected data about the demographics and clinical parameters of migraine severity, COVID-19 infection, vaccination, and characteristics of headaches following vaccination. Out of 841 migraine patients filling in the questionnaire, 66.47% and 60.15% patients experienced a headache attack (from 1 hour to 7 days) after the first and the second vaccine dose, respectively. The main finding concerns headaches perceived by 57.60% of patients: attacks following vaccination were referred to as more severe (50.62% of patients), long-lasting (52.80% of patients) and hardwearing (49.69% of patients) compared to the usually experienced migraine attacks. This could be related to the production of inflammatory mediators such as type Iβ interferon. Considering the high prevalence of migraine in the general population, awareness of the possibility of headaches worsening following COVID-19 vaccination in these patients may allow both patients and clinicians to face this clinical entity with conscious serenity, and to reduce the waste of resources towards inappropriate health-care.

## 1. Introduction

Despite the uncountable measures employed to restrict the spread of COVID-19, the breakthrough in the fight against COVID-19 has been represented by the production and distribution of specific COVID-19 vaccines. Indeed, since 21 December 2020 the European Medicines Agency has approved Comirnaty (Pfizer-BioNTech), an mRNA vaccine, as the first vaccine against SARS-CoV 2 in the European Union. Shortly after, mRNA-1273 (Moderna, 6 January 2021), Vaxzevria (29 January 2021), and Janssen (Ad26.COV2.S—Johnson&Johnson, 11 March 2021) were approved [1]. Although vaccines’ safety and efficacy have been widely documented, randomized controlled trials (RCTs) have reported several systemic reactions in the hours/days following vaccine administrations [2]. Among the various systemic adverse reactions, headache attacks were reported in a high percentage of people after either administration, or both the first and the second [3,4,5,6]. Very recently, the high prevalence of headaches after COVID-19 vaccination with Vaxzevria and Comirnaty has been confirmed in a large population, and clinical features of both acute and persistent headache attributed to COVID-19 vaccination have been defined [7,8,9]. Interestingly, in a post-hoc analysis comparing people with and without primary headaches, patients with pre-existing headaches showed increased attack duration and intensity after COVID-19 vaccination. In line with this observation, in our clinical practice at the University Headache Centre, a high percentage of migraine patients reported headache episodes—described as different from those generally experienced, due to worse pain intensity, attack duration and response to painkillers—in the days following the vaccine administration.

To substantiate the hypothesis of a causative correlation between vaccine administration and worsening of headaches in patients with pre-existing migraine, we aimed to investigate the clinical pattern of headache episodes following COVID-19 vaccine administration (independently of the type of vaccine) in a large sample of headache patients. Furthermore, we analyzed whether the clinical parameters of migraine severity, as well as the type of vaccine administered and/or previous COVID-19 infection, were able to predict the occurrence of headache after COVID-19 vaccine administration in these patients. Finally, we speculated on the putative pathophysiological mechanisms linking COVID-19 vaccination and headache occurrence.

## 2. Materials and Methods

A self-administered, publicly available online questionnaire was developed to collect the following information on patients with migraine: (i) demographic and clinical parameters such as age, diagnosis of previous headaches (migraine without aura, migraine with aura, chronic migraine), disease duration (years), frequency of attacks (headache days/month), ongoing preventive treatments; (ii) data related to previous COVID-19 infection and vaccination, type of vaccine administered and number of doses completed; and finally, (iii) headache episodes occurring in the days immediately following COVID-19 vaccination, focusing on the differences between these headache attacks and those generally experienced by patients in terms of intensity, duration, and response to painkillers. An electronic 15-point questionnaire was created using Google Forms, and administered to 20 non-headache, healthy subjects without a family history of headaches, in order to assess its readability. Subsequently, the questionnaire link was published on Italian Facebook groups oriented to headache patients, with at least 1000 members (such as “Cefalee Campania”, “Forum Emicrania”, “Ho mal di testa”, “Che mal di testa”, “Emicrania Ass. Cefalea Ticino e Le parole dell’emicrania” and, “Alleanza Cefalalgici Al.Ce.”), for 10 days (from 9–19 June). The answers were collected in an online database.

### 2.1. Standard Protocol Approvals, Registrations, and Patient Consents

The study was approved by the local ethics committee of the University of Campania “Luigi Vanvitelli”. The ethics committee board determined that participant consent was not required. The participants were informed that data (i) were collected anonymously in compliance with the recommendations of the ethics committee of the University of Campania “Luigi Vanvitelli” and (ii) due to the anonymization, it was not possible to revoke participation in the study after any answers had been sent.

### 2.2. Statistical Analysis

No statistical power calculation was conducted prior to the study, and the sample size was based on the available data. Categorical data were reported as number and percentage, while continuous data were reported as mean ± standard deviation (SD). We used the chi-square test to compare categorical variables and the independent sample *t*-test to compare continuous variables. Tetrachoric correlation was used to identify the association between dichotomous variables (the occurrence of headaches and other systemic reactions after vaccine administration). Statistical significance was set at *p* < 0.05. A multiple logistic regression analysis was conducted in order to identify whether differences in parameters were found between patients experiencing headache attacks and those who did not, after the vaccine administration; these parameters include the type of vaccine administered, previous COVID-19 infection, and migraine parameters could represent independent predictors of headache attack occurrence after vaccine administration. Finally, a simple binary logistic regression analysis was conducted in order to identify whether the occurrence of a headache attack after the first vaccine administration could represent an independent predictor of headache attack occurrence after the second vaccine administration. All analyses were performed using STATA version 16 (StataCorp, College Station, TX, USA).

## 3. Results

### 3.1. Population

Ten days after the questionnaire was posted on Italian Facebook groups oriented to headache patients, 841 migraine patients filled in the electronic questionnaire. Among these, 553 (65.76%) patients reported migraine without aura, 219 (26.04%) patients reported diagnosis of migraine with aura, 69 (8.20%) patients reported diagnoss of both migraine without aura and migraine with aura. Furthermore, 199 (23.66%) patients reported chronic migraine.

The average frequency of headache attacks (days/month) in the three months before COVID-19 vaccination was >15 in 199 (23.66%) patients, between 9 and 14 in 169 (20.1%) patients, between 5 and 8 in 280 (33.29%) patients and <4 in 193 (22.95%) patients.

Preventive anti-migraine pharmacological treatments were used by 485 (57.67%) patients. Attack/rescue medication were used as follows: 204 (24.26%) patients used triptans, 237 (28.18%) used NSAIDs, 205 (24.38%) alternated between triptans or NSAIDs, 120 (14.27%) patients used a combination of drugs, and only 27 (3.21%) used other painkillers such as opioids, benzodiazepines, steroids or anti-emetic drugs [see Table 1 for further information].

### 3.2. Headache Symptoms after COVID-19 Vaccine in Migraine Patients

Among the whole patient sample, 77 (9.16%) subjects had contracted the COVID-19 infection. All of the patients underwent at least the first administration of the COVID-19 vaccine, and 394 (46.85%) patients also received the second dose (when necessary). A total of 659 (78.36%) patients received the mRNA vaccines (Comirnaty or mRNA-1273) and 182 (21.64%) patients received non-replicating DNA viral vector vaccines (Vaxzevria or Janssen).

**Figure 1:**
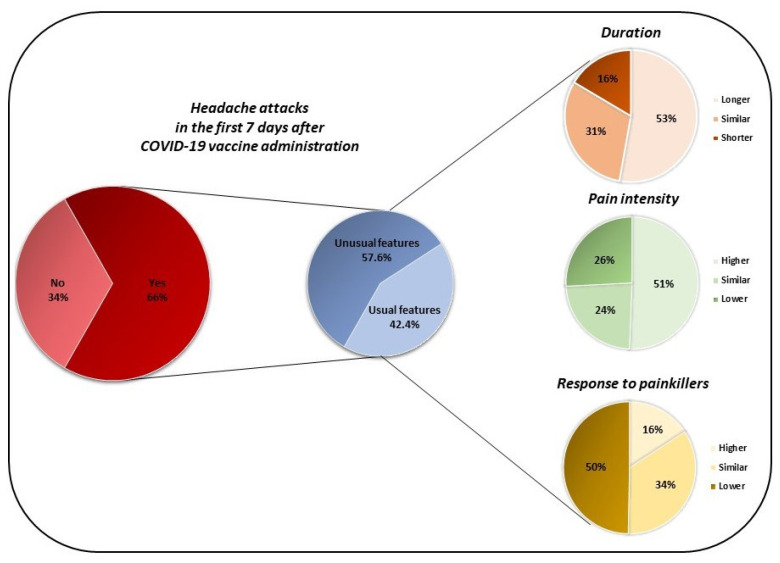
Features of headache attacks occurring in the first 7 days after the first COVID-19 vaccine administration.

According to the questionnaire results, 559 (66.47%) migraine patients experienced at least one headache attack in the period between 1 h and 7 days after the first vaccine administration. More in depth, considering the first vaccine administration, 271 (48.48%) patients experienced attacks in the first 24 h, 165 (29.52%) patients experienced attacks from 24 to 72 h and, finally, 22% (123 patients) reported attacks from 72 h to 7 days. In 322 (57.60%) migraine patients, the attacks following vaccine administration were perceived as “different” compared to those usually experienced, in terms of pain intensity, duration or response to painkiller. Specifically, a higher pain intensity in 163 (50.62%) patients, a longer duration of the headache attacks in 52.80% (170 patients), and a lower responsiveness to painkillers in 49.69% (160 patients) were reported [see Table 2 and Figure 1 for further information].

**Figure 2:**
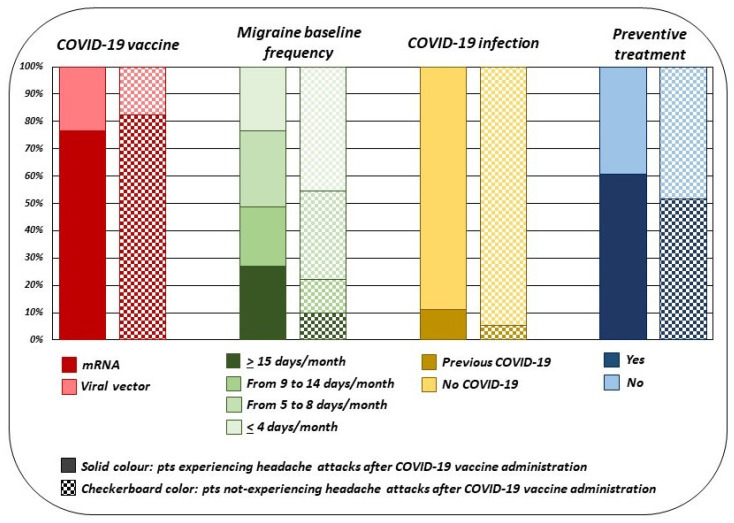
Clinical differences between migraine patients with and without headache attacks after the first COVID-19 vaccine administration.

Among 394 migraine patients (46.85%) receiving both vaccine doses, 237 patients (60.15%) experienced at least one headache attack between 1 h to 7 days after the second vaccine administration. More in depth, considering the second vaccine administration, 51.9% (123 patients) experienced attacks in the first 24 h, 32.07% (76 patients) experienced attacks from 24 to 72 h and, finally, 16.03% (38 patients) reported an attack from 72 h to 7 days. In 130 (54.85%) migraine patients, the attacks following the vaccine administration were perceived as “different” compared to those usually experienced; specifically, a higher pain intensity in 74 patients (56.92%), a longer duration of the headache attacks in 66 patients (50.77%), and a lower responsiveness to painkillers in 69 patients (53.08%) were reported. When compared to migraine patients not experiencing headache attacks, migraine patients experiencing headache attacks after the first vaccine administration showed higher baseline frequency of migraine days per month (*p* < 0.001), prevalence of both ongoing preventive anti-migraine pharmacological treatments (*p* < 0.001), and previous COVID-19 infection (*p* = 0.04). Furthermore, headache attacks after COVID-19 vaccination were significantly less frequent in patients receiving mRNA vaccines (Comirnaty or mRNA-1273) compared to patients receiving non-replicating DNA viral vector vaccines (Vaxzevria or Janssen) (*p* = 0.03) (see Figure 2).

Finally, among 219 patients suffering from migraine with aura, 57.08% (125 patients) and 59.18% (58 patients) reported an attack between 1 h to 7 days after the first and second vaccine administration, respectively. The logistic regression analysis showed that a model including baseline frequency of headache attacks, concomitant migraine preventive treatment administration, types of COVID-19 vaccines administered, and previous COVID-19 infection, is able to predict the occurrence of headache attacks after the first COVID-19 vaccination [χ2(4) = 28.76, *p* < 0.001]. Odds ratio analysis demonstrated that age (odds = 0.98 *p* = 0.01), frequency of headache attacks (odds = 1.25 *p* = 0.001) and previous COVID-19 infection (odds = 2.13 *p* = 0.01) were associated with an increased likelihood of experiencing headache attacks after the first COVID-19 vaccine administration.

### 3.3. Post-Hoc Sub Analyses

Considering a cut-off of 55 years (in line with previous RCTs and real-life observations) [3,4,5], no difference was found in the percentage of patients experiencing headache attacks after the first COVID-19 vaccine administration. Similarly, no significant difference was found in the percentage of patients reporting headache attacks after the first and the second COVID-19 vaccinations. Correlation analysis showed a statistically significant correlation between the occurrence of headache attacks in the days following the first vaccine administration and the other systemic adverse reactions (r = 0.33, *p* < 0.001). On the other hand, the presence of a headache attack after the first vaccine administration was significantly associated with the occurrence of an attack after the second vaccine administration [logistic regression analysis χ2(4) = 59.40, *p* < 0.001 odds = 5.33 *p* < 0.001].

## 4. Discussion

In the present study, we investigated the incidence of headache attacks in the hours or days following COVID-19 vaccination in patients with migraine. We found that, among patients filling in the online questionnaire, 559 patients (66.47%) experienced headache attacks between 1 h and 7 days after the first vaccine administration, while 237 patients (60.15%) experienced headache attacks between 1 h and 7 days after the second administration. Interestingly, over the half of patients perceived the headache attacks as “different” from those usually experienced. Indeed, patients described those attacks as characterized by higher pain intensity, longer duration and reduced responsiveness to usually effective painkillers. The safety and efficacy of COVID-19 vaccines have been demonstrated by phase 3 RCTs. Among these, Comirnaty and Vaxzevria trials showed good efficacy (e.g., immunization) (95% and 99%, respectively) with safety profiles similar to previous viral vaccines, except for the higher prevalence of local reactions (e.g., erythema, induration and tenderness) and systemic events (e.g., fatigue, headache, fever, myalgia and arthralgia); these were reported more frequently after the second vaccine administration, and more frequently in younger (16–55 years old) than in older people (>55 years old), as well as in subjects with previous COVID-19 infection (see Table 3 for further information). Among the systemic reactions described using different COVID-19 vaccine RCTs, headache incidence increased from 32.7 to 65% after the first administration, and from 31 to 60.2% after the second administration [3,4,5,6]. However, neither data about headache features nor about headache incidence in patients experiencing pre-existing primary headaches were available, considering the very high prevalence of these conditions, specifically migraine, in the general population.

Very recently, real-world observations shed light on the clinical features of headache attacks following Vaxzevria or Comirnaty administrations, delineating the profile of “acute headache attributed to COVID-19 vaccination with the ChAdOx1 nCoV-19 (AZD1222) vaccine” [7,8]. According to the proposed criteria, headache attacks occur within 24 h following COVID-19 vaccinations, and last up to 72 h before resolving, generally in parallel with stabilization or improvement of other systemic manifestations (fatigue, chills, exhaustion or fever). The headache phenotype is migraine-like, characterized by being pressing or dull in quality, of moderate or severe intensity, and associated with phonophobia and photophobia. However, despite the very large cohort of subjects, a pre-existing primary headache was reported only in a percentage of patients, where the headaches following COVID-19 vaccinations were reported as longer and more severe compared to those experienced by subjects with no history of primary headaches. [7,8] These data are partially in line with our findings, showing a very high prevalence of patients with migraine experiencing headache attacks following the first (559/841 migraine patients—66.47%) or second (237/394 migraine patients—60.15%) COVID-19 vaccination, and independently of the types of vaccine administered. Among these, it is noteworthy that a very high percentage of patients (322/559 migraine patients—57.60%) perceived the headache attacks as more painful and long lasting compared to those usually experienced before the COVID-19 vaccination. Interestingly, data arising from the questionnaire highlighted that the headaches following the first or second COVID-19 vaccination were less responsive to usually effective painkillers, such as NSAID, triptans, analgesics in combination, opioids, benzodiazepines, steroids or anti-emetic drugs. The reason for which COVID-19 vaccine administration can lead to more severe, long-lasting and hardwearing headache attacks in migraine patients is unknown, considering that data about headaches as adverse effects of different types of viral vaccines (e.g., influenza vaccines) are not available. However, starting with vaccine-induced immunization mechanisms, several processes can be invoked. Vaccines currently approved or under scrutiny in humans are developed using one of two different platforms: non-replicating DNA viral vectors or mRNA [9,10]. The non-replicating DNA viral vectors (e.g., chimpanzee adenovirus) work as carriers of a gene coding for the viral spike protein that induce the production—via mRNA—of the COVID-19 spike proteins, subsequently migrating to the cell surface. Between a few hours and 1 day post-vaccination the host’s immune system recognizes spike proteins, and ignites the immune reactions by engaging multiple pattern-recognition receptors and inducing type I interferon secretion, with consequent delivery of both antigenic and inflammatory signals to T cells in the lymph nodes [11]. On the other hand, RNA vaccines using the host cell transcription machinery [9,10] serve as both immunogen (e.g., encoding the viral protein) and adjuvant (e.g., owing to intrinsic immunostimulatory properties of RNA). More specifically, mRNA is recognized by endosomal and cytosolic innate sensors in the cells, resulting in the activation and production of multiple inflammatory mediators such as type Iβ interferon (IFN-1β) [12,13]. Interestingly, clinical evidence coming from IFN-1β treatments supports its critical role in both migraine occurrence or worsening, and “flu-like symptoms” such as fatigue, chills and fever [14,15,16,17,18]. The IFN-1β migraine-promoting effect may be related to the imbalance between decreased TNF-α and IFN-γ (e.g., CD3-mediated type 1 helper cell inflammatory cytokines) along with IL-4 and IL-10, and increased IL-6 production, a well-known inducer of acute-phase protein expression (e.g., T-cell-activating inflammatory cytokines via IL-2 induced proliferation and differentiation of CD4 + T cells). Nevertheless, it cannot be excluded that the IFN-1β migraine-promoting effect may also be sustained by the activation of nuclear factors—such as the kappa-light-chain enhancer of activated B-cells (NF-kB)—in the trigeminal system, with secondary effects on the nitric oxide pathway [19]. Furthermore, a recent study supported the IFN-1β contribution in enhancing neocortical hyper-excitability [20]. Interestingly, it is noteworthy that altogether, IL-6, nitric oxide pathway and cortical dis-excitability have been recognized as a critical moment in migraine pathophysiology [21,22].

Based on data collected from COVID-19 vaccine RCTs, showing an increased incidence of systemic reactions after the second vaccine administration, an enhanced inflammatory response has been argued; this is likely related to the short-term changes of innate cells, such as macrophages (so-called ‘trained immunity’) or, alternatively, the activation of memory T and B cells generated after the first vaccine injection. However, in the present study, we found no differences in headache attack incidence in the 7 days following the first vaccine administration, compared to the 7 days following the second, in migraine patients who received both vaccines doses. While the incidence of headache episodes after the second dose is in line with what emerges from the RCTs, the higher incidence of attacks after the first administration is surprising. We cannot achieve a comprehensive explanation of the higher incidence of migraine attacks after the first COVID-19 vaccine administration (leading to a super-imposable incidence of migraine attacks in the second COVID-19 vaccine administration) compared to RCT data. Nevertheless, it is widely recognized that migraine can be deciphered as a genetically determined brain state, making patients prone to produce headaches in response to actual or putative threatening conditions (e.g., maladaptive stress, homeostatic abnormalities, environmental changes, use or withdrawal of some substances, infections, etc.) [23,24]. In line with this interpretation, we may speculate that headaches experienced by migraine patients after COVID-19 vaccine administration may represent a heightened brain reaction to a significant systemic, vaccine-induced, immune-mediated activation; this is usually observed in migraine patients as a consequence of various inflammatory states acting on modulation of the pain threshold, trigeminal nerve fiber sensitization, and ultimately, the precipitation of migraine [25].

Focusing on two different vaccine platforms (e.g., non-replicating DNA viral vectors or mRNA), our post-hoc analysis showed that the percentage of migraine patients reporting headache attacks following COVID-19 vaccine administration was significantly higher in patients receiving non-replicating DNA viral vectors vaccines (Vaxzevria or Janssen), compared to those receiving mRNA vaccines (Comirnaty or mRNA-1273) (*p* = 0.03). Based on the assumption that migraine attacks may represent the brain’s reaction to systemic immune-mediated inflammation, we wonder about the possibility that additional mechanisms could be invoked to explain the higher percentage of migraine attacks in patients receiving non-replicating DNA viral vectors vaccines. In order to clarify this issue, we can take inspiration from the pathogenetic mechanisms proposed to clarify the thrombosis with thrombocytopenia syndrome recently reported after vaccination with Vaxzevria. It is noteworthy that the interaction between platelets’ ACE2 receptor and free-floating spike proteins (released in a large amounts when a vaccinated cell dies or is destroyed by the immune system) can promote ACE2 internalization and degradation. The loss of ACE2 receptor activity leads to a reduced angiotensin-II inactivation (with an increased angiotensin-II activity), and consequently, a reduced generation of angiotensin-(1–7), both able to trigger inflammation, platelet aggregation and thrombosis [26]. Within this framework, angiotensin II, through its receptors, induces mast cell degranulation, resulting in meningeal irritation and stimulation of nociceptive trigeminal nerve endings; these are mechanisms known to be involved in migraine ignition.

Furthermore, angiotensin-II may also modulate the effects of the peptidergic systems involved in neurogenic inflammation and nociceptive transmission, where a relevant role is played by substance-P, a regulator of sensory transmission in the trigeminal and other dorsal root ganglia [27]. The effects of the COVID-19 vaccine on platelet aggregation may be further supported by the high occurrence of migraine with aura attacks following vaccine administration [28]. Indeed, an increase in the release of platelet-dense bodies, containing glutamate, along with a low threshold of platelet activity, have been invoked among the mechanisms underpinning the aura phenomenon in people with MwA [29,30]. Interestingly, our secondary analysis did not show differences in the incidence of headache attacks following vaccine administration in under 55 year-old patients compared to over 55 year-old patients. The data are not in line with those emerging from RCTs showing a higher occurrence of systemic reactions in patients aged <55 years; this is probably due to aging-related differences in the magnitude of immune system activity (e.g., loss of T cell receptor diversity in both CD8 and CD4 cells, reduced T cell survival, favored production of short-lived effector T cells over memory precursor cells) [31,32,33,34]. It can be argued that the magnitude of immune system activation observed in the elderly is less able to induce systemic reactions (including headache attacks) in subjects with no history of migraine, whereas it is enough to ignite headache attacks in the peer patients with migraine. The logistic regression analysis showed that the clinical parameters able to predict the occurrence of an attack after the first vaccine administration were a high baseline frequency of migraine attacks, the intake of migraine preventive therapies, previous COVID-19 infection and the viral vector vaccine rather than mRNA. This finding suggests that a more severe clinical phenotype (characterized by a higher frequency of attacks and use of migraine preventive treatments) represents a risk factor for migraine attacks following vaccine administration. On the other hand, COVID-19 infection could represent a predictive factor, as it is associated with a heightened response of the immune system due to the previously described immune memory mechanisms. Finally, it is noteworthy that the occurrence of a headache attack after the first vaccine administration is significantly associated with the occurrence of an attack after the second vaccine administration, a relevant finding considering the high possibility of further booster doses.

We are aware that our study is not exempt from some limitations. First of all, due to the fact that the questionnaire was filled in by patients with migraine from Facebook groups known to engage patients experiencing more severe phenotypes, the sample under examination does not reflect the general migraine population in terms of disease severity (e.g., percentage of chronic migraine, preventive treatments, monoclonal antibody therapies, etc.). Nevertheless, the digital questionnaire gave us the possibility to obtain data from a large number of patients in a brief time frame. Furthermore, considering the well-known role of emotional stress in migraine attacks, we cannot exclude that the concerns associated with COVID-19 vaccine administration may have worked as trigger factors for headache attacks worsening. Finally a control group is missing.

Finally, according to the IHS classification (ICHD-3), “when a pre-existing primary headache is made significantly worse (usually meaning a two-fold or greater increase in frequency and/or severity) in close temporal relation to such a causative disorder, both the primary and the secondary headache diagnoses should be given, provided that there is good evidence that the disorder can cause headache”. [35] Therefore, we support that “COVID-19 vaccine administration headache” could represent a new nosological entity to be included in the ICHD-3.

Considering both the high prevalence of such a disabling disorder as migraine (about 12–14% of the general population [36]) and the even higher prevalence of COVID-19 vaccine administration all over the world, we should be aware that several millions of patients with migraine could be concomitantly burdened by high disability for a certain period of time.

Moreover, spreading the awareness of possible headache worsening following COVID-19 vaccine administration in migraine patients may allow clinicians to face this condition with conscious serenity, and to reduce the waste of resources towards inappropriate health-care. On the other hand, patients with migraine should be made aware and reassured about the possibility of increased headache severity following vaccine administrations.

## Figures and Tables

**Table 1:** Baseline characteristics of migraine patients filling in the questionnaire (841 pts).

	Baseline % (n)
Age (mean years ± SD)	44.94 ± 12.13
Disease history (mean years ± SD)	25.45 ± 13.93
Diagnosis	
Migraine with aura	26.04% (219)
Migraine without aura	65.76% (553)
Both migraine with aura and migraine without aura	8.20% (69)
Baseline headache days/month	
<4	22.95% (193)
5–8	33.29% (280)
9–14	20.10% (169)
>15	23.66% (199)
Pts taking preventive treatment	57.67% (485)
Painkillers	
Triptans	24.26% (204)
Acetaminophen	4.99% (42)
NSAIDs	28.18% (237)
Triptans or NSAIDs	24.38% (205)
Combination drugs	14.27% (120)
Others	≈1%
Nothing	≈1%
Pts with previous COVID-19	9.16% (77)
Vaccine administered	
Comirnaty	67.30% (566)
Vaxzervria	19.50% (164)
mRNA-1273	11.06% (93)
Janssen	2.14% (18)
Pts completed both vaccine doses	46.85% (394)

SD: standard deviation.

**Table 2:** Clinical features of headache attacks and local/systemic adverse reactions occurring after COVID-19 vaccine administrations.

	After the First Dose (841 pts) % (n)	After the Second Dose (394 pts) % (n)
**Pts with headache after vaccination**	66.47% (559)	60.15% (237)
From 1 to 24 h	48.48% (271)	51.9% (123)
From 24 h to 3 days	29.52% (165)	32.07% (76)
From 3 to 7 days	22% (123)	16.03% (38)
**Headache with different features**	57.6% (322)	54.85% (130)
**(compared to baseline)**		
**Attack duration**		
Greater	52.80% (170)	50.77% (66)
Similar	30.75% (99)	30% (29)
Lower	16.46% (53)	19.23% (25)
**Pain intensity**		
Greater	50.62% (163)	56.92% (74)
Similar	23.6% (76)	21.54% (28)
Lower	25.78% (83)	21.54% (28)
**Response to painkillers**		
Greater	15.84% (51)	13.85% (18)
Similar	34.47% (111)	33.08% (43)
Lower	49.69% (160)	53.08% (69)
**Pts with other adverse reactions**	74.55% (627)	73,60% (290)
Fever	18.79% (158)	27.66% (109)
Pain at the injection site	58.15% (489)	51.52% (203)
Fatigue	31.63% (266)	40.86% (161)
Diarrhoea	4.76% (40)	7.10% (28)
Myalgia	32.10% (270)	45.94% (181)
Others	7.85% (66)	8.12% (32)
**Onset of side effects after vaccination**		
From 1 to 24 h	57.58% (361)	59.31% (172)
From 24 h to 3 days	33.01% (207)	33.10% (96)
From 3 to 7 days	9.41% (59)	7.59% (22)

**Table 3:** Most common local and systemic adverse reactions of COVID-19 vaccines in general population from RCTs, and in migraine patients, from the online questionnaire.

	Comirnaty	mRNA-1273	Vaxzevria	Janssen	Migraine Patients
**Injection site pain** (after the first dose)	83% (16–55) 71% (>55)	84.2%	16% (18–55) 3% (56–69) 6% (>70)	48.6%	58.26%
**Injection site pain** (after the second dose)	78% (16–55) 66% (>55)	88.6%	14% (18–55) 3% (56–69) 0% (>70)		51.52%
**Headache** (after the first dose)	42% (16–55) 25% (>55)	32.7%	65% (18–55) 50% (56–69) 13% (>70)	38.9%	66.47%
**Headache** (after the second dose)	52% (16–55) 39% (>55)	58.6%	31% (18–55) 34% (56–69) 9% (>70)		60.15%
**Fatigue** (after the first dose)	47% (16–55) 34% (>55)	37.2%	76% (18–55) 50% (56–69) 30% (>70)	38.2%	31.75%
**Fatigue** (after the second dose)	59% (16–55) 51% (>55)	65.3%	55% (18–55) 41% (56–69) 20% (>70)		40.96%
**Myalgia** (after the first dose)	21% (16–55) 14% (>55)	22.7%	53% (18–55) 37% (56–69) 11% (>70)	33.2%	31.27%
**Myalgia** (after the second dose)	37% (16–55) 29% (>55)	58%	35% (18–55) 24% (56–69) 11% (>70)		45.18%
**Fever** (after the first dose)	4% (16–55) 1% (>55)	0.8%	24% (18–55) 0% (56–69) 4% (>70)	9%	18.79%
**Fever** (after the second dose)	16% (16–55) 11% (>55)	15.5%	0% (18–55) 0% (56–69) 0% (>70)		27.66%

## Data Availability

All data generated or analysed during this study are available from the corresponding author (dottor.russo@gmail.com).
